# Handheld infrared thermography for triage of LVAD driveline infection: A prospective pilot diagnostic accuracy study

**DOI:** 10.1016/j.jhlto.2026.100613

**Published:** 2026-06-15

**Authors:** Theofanis Mavrepis, Stijn Legtenberg, Peter van der Meer, Michiel Kuijpers, Yvonne Douglas, Michiel E. Erasmus, Jozine M. ter Maaten, Stan A.J. van den Broek, Jan A. Krikken, Kevin Damman

**Affiliations:** a University of Groningen, Departement of Cardiology, University Medical Center Groningen, Groningen, The Netherlands; b University of Groningen, Departement of Cardiothoracic Surgery, University Medical Center Groningen, Groningen, The Netherlands

**Keywords:** Left ventricular assist device, Driveline infection, Infrared thermography, Diagnostic accuracy, Triage

## Abstract

**Background:**

Existing approaches to diagnose driveline infections (DLI) in patients with durable left ventricular assist devices (LVADs) differ in speed, cost, and specificity. Handheld infrared thermography may support DLI through rapid, easy, non-contact triage by detecting local temperature elevation.

**Methods:**

In this prospective, single-center diagnostic accuracy study, adult LVAD patients underwent handheld thermal imaging of the driveline exit site. Performance of thermography in predicting clinically diagnosed DLI was assessed using receiver operating characteristic analysis (AUC with 95% confidence intervals [CI], DeLong). The threshold derived using Youden’s J was considered exploratory and intended for internal within-study performance estimation only; sensitivity, specificity, PPV, and NPV were calculated with exact (Clopper-Pearson) 95% CIs. Associations were assessed using logistic regression and Firth’s penalized logistic regression; correlation with high-sensitivity C-reactive protein was assessed using Spearman’s ρ.

**Results:**

Fifty patient episodes were analyzed, of which 6 (12%) had DLI. Median peri-driveline temperature was higher in patients with DLI than without DLI (36.1°C vs 34.4°C; *p* = 0.005). Peri-driveline temperature demonstrated good discrimination for DLI (AUC 0.86, 95% CI 0.72-1.00). The threshold of 34.7°C yielded sensitivity 100% (95% CI 54-100), specificity 61% (95% CI 45-76), PPV 26% (95% CI 10-48), and NPV 100% (95% CI 87-100). Each 1°C increase in temperature was associated with higher odds of DLI in Firth regression (OR 3.7, 95% CI 1.4-14.5; *p* = 0.006). Temperature and hs-CRP were weakly correlated (ρ = 0.11; *p* = 0.45). Sensitivity analysis, including a second infection episode, produced similar discrimination.

**Conclusions:**

Handheld infrared thermography showed good discrimination for DLI, and no false negatives were observed at a low threshold, albeit with limited specificity. These findings support further evaluation as a triage adjunct rather than a stand-alone diagnostic test.

## Background

Durable mechanical circulatory support with a left ventricular assist device (LVAD), particularly with contemporary continuous-flow devices, is an established treatment for selected patients with advanced heart failure.^1^, ^2^, ^3^, ^4^ As LVAD utilization expands and duration of device therapy increases, device-related complications have become increasingly clinically relevant. Driveline infection (DLI) is among the most frequent and impactful complications and is associated with recurrent hospital admissions, risk of sepsis, and the need for surgical revision in selected cases.^5^, ^6^, ^7^, ^8^, ^9^ Management of DLI often requires prolonged, and in some cases lifelong, antimicrobial therapy, highlighting its chronic and burdensome nature. In advanced or refractory cases, DLI may even necessitate LVAD exchange and can adversely affect transplant eligibility, thereby directly negatively influencing long-term prognosis. Established risk factors for DLI include driveline trauma, higher body mass index, diabetes mellitus, and other patient- and device-related factors.^9^, ^10^, ^11^

Early recognition of DLI can be challenging because local findings may be subtle and can overlap with non-infectious irritation or chronic exit-site changes.^5^, ^7^ Inflammatory markers (e.g., high sensitive c reactive protein (hs-CRP), leukocyte count) can support clinical suspicion but are non-specific and may not reflect a localized process.^12^ FDG-PET/CT can identify deeper or occult infectious foci in LVAD patients but is resource-intensive and impractical for frequent exit-site monitoring.^13^

Infrared thermography maps skin surface temperature by detecting emitted infrared radiation. Because inflammation and infection may increase local perfusion and surface temperature, thermography has been explored in cellulitis and wound (surgical or other) monitoring, often with high sensitivity but more limited specificity.^14^, ^15^, ^16^, ^17^, ^18^, ^19^ Handheld thermography devices are portable, simple to operate and fast, making them an attractive potential adjunct for triage in LVAD outpatient care. However, the diagnostic accuracy of handheld thermography for DLI has not been established.

We therefore conducted a prospective single-center diagnostic accuracy study to evaluate whether quantitative peri-driveline temperature from handheld infrared imaging can discriminate between LVAD patients with and without DLI and to explore the relationship between peri-driveline temperature and CRP.

## Methods

### Study design and setting

This prospective, single-center diagnostic accuracy study was conducted at the University Medical Center Groningen between April and June 2025. All patients provided written informed consent for inclusion in an ongoing observational biobank (CardioLines-LVAD (local ethical committee approval: METc 2012/296)). Reporting follows STARD 2015.^20^

### Participants

Adults (≥18 years) with a durable LVAD (HeartMate 3) who provided written informed consent for the biobank were eligible. Participants were included consecutively during routine outpatient visits and during hospital admission for suspected or confirmed DLI. There were no disease-specific exclusions.

### Diagnosis of DLI

The reference diagnosis was the treating cardiology team’s final clinical diagnosis of DLI, based on the totality of available clinical information, including local exit-site findings, microbiology from wound and/or blood cultures, laboratory findings, including hs-CRP, imaging such as FDG-PET/CT when clinically indicated, and subsequent clinical course. Thermal imaging results were not used to establish the clinical reference diagnosis. DLI-positive episodes were additionally mapped retrospectively using ISHLT compatible infection categories and summarized in Supplementary Table 3.^21^, ^22^

### Image acquisition

Thermal images were acquired with a Lodestar LTi120S handheld infrared thermal imager (spectral range 8-14 μm). Dressings and clothing were removed, and imaging was performed under routine clinical conditions without direct sunlight. Where feasible, patients were imaged supine; wheelchair users were imaged seated. The camera was held approximately 40 cm from the exit site and oriented perpendicular to the skin surface. Exact camera-to-skin distance and imaging angle were not formally measured or recorded. Systematic core or peripheral body thermometer measurements were not obtained at the time of thermographic imaging. Timing relative to clinical care processes (including potential antibiotic initiation in admitted patients) was not standardized. Ambient temperature and patient activity before imaging were not systematically recorded. Patients included in the primary analysis underwent a single thermographic assessment. All images were acquired by a single investigator; interobserver and intraobserver variability were not formally assessed. All images were acquired by a single investigator. Interobserver variability was not assessed; however, repeated blinded ROI placement by the same reader was performed as an intra-reader consistency assessment.

### Image processing, calibration, and temperature extraction

**Figure 1:**
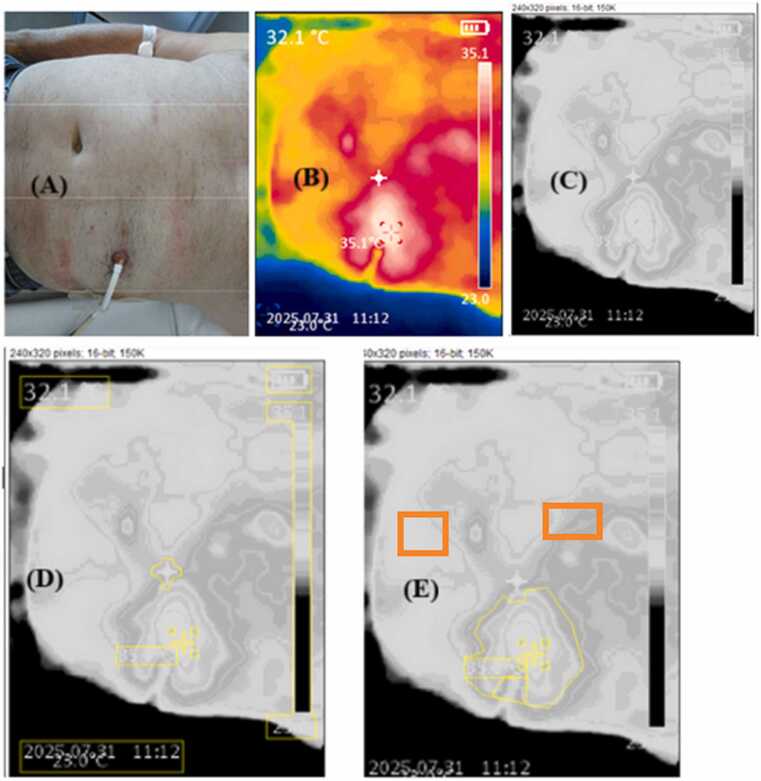
*Visualization of the image analysis process.* All images were acquired from the abdominal area of a patient with a driveline infection (DLI). (A) Standard photograph used for anatomical orientation. (B) Infrared image exported in bitmap (BMP) format from the infrared camera. (C) BMP infrared image after import into Fiji/ImageJ for image processing. (D) Masking of camera-generated graphics and non-skin artifacts prior to temperature extraction. (E) Final manually delineated peri-driveline tissue region of interest (yellow), excluding the driveline cable and graphical overlays, used for mean peri-driveline temperature extraction after image calibration. The 2 abdominal reference-skin regions of interest used for the exploratory ΔT analysis are shown in orange.

Thermal images were exported at full sensor resolution and analyzed in Fiji/ImageJ version 2.14.0^23^ using a pre-specified workflow defined before linkage to the clinical reference diagnosis. On-screen camera overlays were masked. Pixel intensities were converted to °C using the frame-specific displayed minimum and maximum temperatures, enabling consistent within-image extraction but not external validation of absolute temperature accuracy. A peri-driveline skin region of interest (ROI) was manually delineated around the exit site using priori rules, excluding the driveline cable, dressings, visible artifacts, and non-skin regions. The primary index-test variable was mean ROI temperature (peri-driveline temperature), pre-specified to reduce sensitivity to focal hotspots, single-pixel noise, and minor ROI edge variation. ROIs were drawn using coded study identifiers only; temperature values were merged with the reference diagnosis after completion of ROI delineation and extraction for the full dataset. The image acquisition process is visualized in Figure 1.

As an exploratory, non-prespecified relative-temperature analysis, 2 abdominal reference-skin ROIs were additionally placed on skin regions away from the driveline exit site and visible local artifacts. ΔT was defined as the mean peri-driveline ROI temperature minus the mean temperature of the 2 abdominal reference-skin ROIs. The abdominal reference-skin ROIs were used as relative skin-temperature comparators and were not intended to represent measured core or peripheral body temperature.

### Blinding

Thermal images were coded with study identifiers prior to analysis. ROI delineation and temperature extraction were performed using a pre-specified processing approach (Supplementary Appendix) described in the paragraph above, without referencing the final clinical classification. Temperature outputs were linked to the reference diagnosis only after extraction for statistical analysis.

### Statistical analysis

Continuous variables are reported as mean (standard deviation (SD)) when normally distributed or as median [IQR; 25th-75th percentile] otherwise. Between-group comparisons were performed using Welch’s *t* test for approximately normally distributed variables and the Mann-Whitney *U* test for non-normal variables. Categorical variables are reported as counts (percentages) and compared using Fisher’s exact test.

AUCs are reported with 95% confidence intervals estimated using the DeLong method, appropriate for rank-based receiver operating characteristic (ROC) statistics. At the selected threshold using Youden's J, sensitivity, specificity, PPV, and NPV were calculated from the confusion matrix, with exact (Clopper-Pearson) 95% confidence intervals for binomial proportions. We intentionally prioritized sensitivity to explore a rule-out strategy; thresholds should be considered provisional.

To assess internal stability of the primary peri-driveline temperature ROC analysis and threshold selection, we performed stratified non-parametric bootstrapping with 2000 resamples, preserving the number of DLI-positive and DLI-negative observations in each resample. In each bootstrap sample, the ROC curve was reconstructed, and the Youden-derived threshold was re-estimated. Bootstrap distributions of AUC and selected thresholds were summarized using medians and 2.5th to 97.5th percentiles. Because of the small number of DLI events, this analysis was interpreted as an internal stability assessment rather than validation. As an additional influence analysis, the ROC analysis was repeated after sequential exclusion of each DLI-positive episode.

Associations between peri-driveline temperature and DLI were assessed using univariable logistic regression. Given the low number of events and risk of separation, Firth’s penalized logistic regression was also applied. A multivariable Firth model including peri-driveline temperature and hs-CRP was fitted to assess whether temperature remained associated with DLI after accounting for hs-CRP (complete cases only). Correlation between peri-driveline temperature and hs-CRP was assessed using Spearman’s ρ. A pre-specified sensitivity analysis included a second infection admission from the same patient (patient-episode analysis) and repeated ROC and regression analyses.

The exploratory ΔT analysis was analyzed analogously to the primary index-test analysis. ΔT distributions were compared between DLI-positive and DLI-negative patients using the Mann-Whitney *U* test. The apparent AUC was estimated with 95% confidence intervals using the DeLong method. Diagnostic performance at the exploratory Youden-derived ΔT threshold was calculated from the confusion matrix, with exact Clopper-Pearson 95% confidence intervals for sensitivity, specificity, PPV, and NPV. A stratified non-parametric bootstrap internal stability assessment with 2,000 resamples was performed for the ΔT AUC and selected threshold. An episode-level ΔT sensitivity analysis included the second infected episode and applied the primary patient-level ΔT threshold.

No formal sample-size calculation was performed because this was a prospective pilot diagnostic-accuracy study embedded in a pragmatic clinical imaging workflow. The sample size was determined by the number of eligible LVAD patients available during the predefined inclusion period. Diagnostic performance estimates are therefore reported with confidence intervals and interpreted as exploratory.

Analyses were conducted only on available cases without imputation. There were no patient exclusions due to low-quality thermal images. Two tailed *p*-values < 0.05 were considered statistically significant. All statistical analyses were performed using R version 4.5.0 (R Foundation for Statistical Computing, Vienna, Austria).

## Results

### Participants

In total, 50 unique patient episodes (50 patients total) were included in the primary analysis, of which 6 (12%) episodes were classified as DLI. In the overall cohort, the mean age was 56.2 ± 10.8 years, 20% of patients were female, and LVAD indication was bridge-to-decision in 30%, bridge-to-transplant in 26%, and destination therapy in 44%. Antibiotics were being used at imaging in 17/50 patients, of which 11/44 DLI-negative patients. Baseline characteristics of the included patient episodes are summarized in Table 1. Antibiotic indications, antibiotic types, and exploratory DLI-negative subgroup characteristics are summarized in Supplementary Tables 6 and 7

**Table 1:** Patient Baseline Characteristics

**Variable**	**All patients (*n* = 50)**	**No DLI (*n* = 44)**	**DLI (*n* = 6)**	***p*-value**
Age (years)	56.2 (10.8)	56.0 (11.2)	57.0 (8.3)	0. 812
Male	40 (80.0)	34 (77.3)	6 (100)	**-**
BMI (kg/m²)	27.6 (3.8)	27.4 (3.9)	29.2 (2.7)	0.183
Type 2 diabetes	9 (18)	8 (18.2)	1 (16.7)	1.000
*LVAD characteristics*				
Years since LVAD	2.5 [1.5, 4.4]	2.5 [1.5, 4.1]	3.0 [1.3, 5.5]	0.904
Bridge to decision	15 (30.0%)	13 (29.5%)	2 (33.3%)	**-**
Bridge to transplant	13 (26.0%)	11 (25.0%)	2 (33.3%)	-
Destination therapy	22 (44.0%)	20 (45.5%)	2 (33.3%)	-
*Laboratory values*				
Hb (mmol/L)	8.8 (0.8)	8.9 (0.8)	8 (1.0)	0.099
Leukocytes (10⁹/L)	8.0 [7.1, 9.1]	8.0 [7.2, 9.25]	6.90 [6.1, 7.92]	0.088
hs-CRP (mg/L)	4.5 [1.6, 8.8]	3.6 [1.5, 8.0]	20 [6.5, 34.3]	0.008

BMI, Body Mass Index; Hb, Hemoglobin; hs-CRP, high-sensitivity C-reactive protein; IQR, Interquartile Range; LVAD Left Ventricular Assist Device; n, number; SD, Standard Deviation.

### Comparison between groups

**Figure 2:**
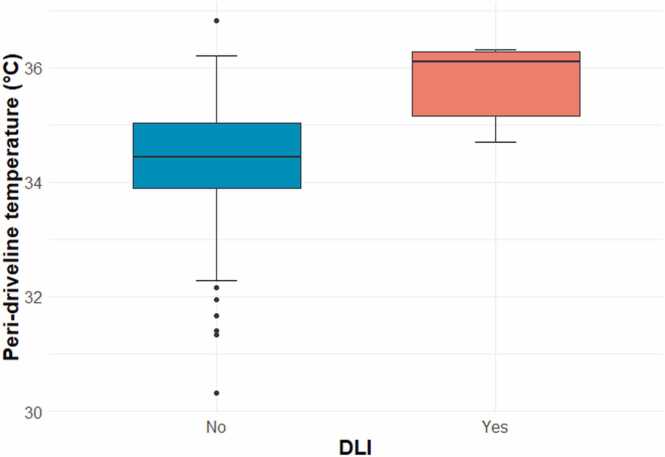
*Distribution of the peri-driveline temperature by infection status.* Boxplot showing peri-driveline temperature measurements in patients with and without confirmed driveline infection (DLI).

**Figure 3:**
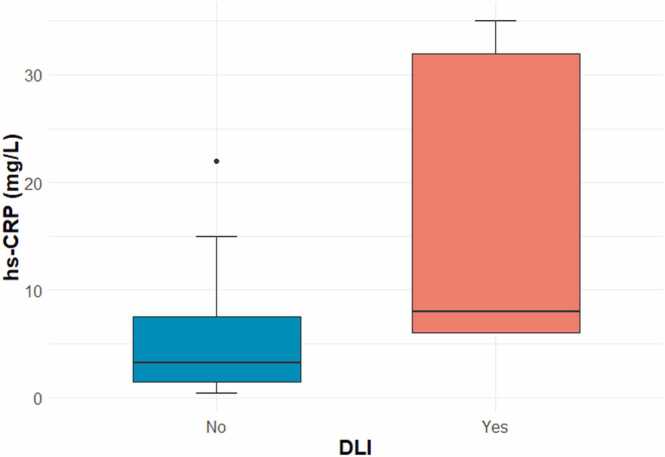
*Distribution of hs-CRP by infection status.* Boxplot showing hs-CRP measurements in patients with and without confirmed driveline infection (DLI). Values >50 mg/L were excluded from this boxplot for visual purposes (96 mg/L in infection; 127 mg/L in no infection).

Patients with a DLI had significantly higher peri-driveline skin temperatures than patients without DLI (36.1°C [IQR 35.2-36.3] vs 34.4°C [34.0-35.0]; *p* = 0.0048) (Table 2; Figure 2). Similarly, hs-CRP concentrations were higher in the DLI group (20.0 mg/L [6.5-34.3] vs 3.6 mg/L [1.5-8.0]; *p* = 0.008) (Figure 3).

**Table 2:** Average Peri-Driveline Temperature

Variable	All patients (*n* = 50)	No Infection (*n* = 44)	Infection (*n* = 6)	*p*-value
Peri-driveline Temperature(°C) (median (IQR))	34.7 [33.9, 35.3]	34.4 [33.9, 35.0]	36.01 [35.2, 36.3]	0.0048

IQR, Interquartile Range; n, number.

### Diagnostic accuracy

**Figure 4:**
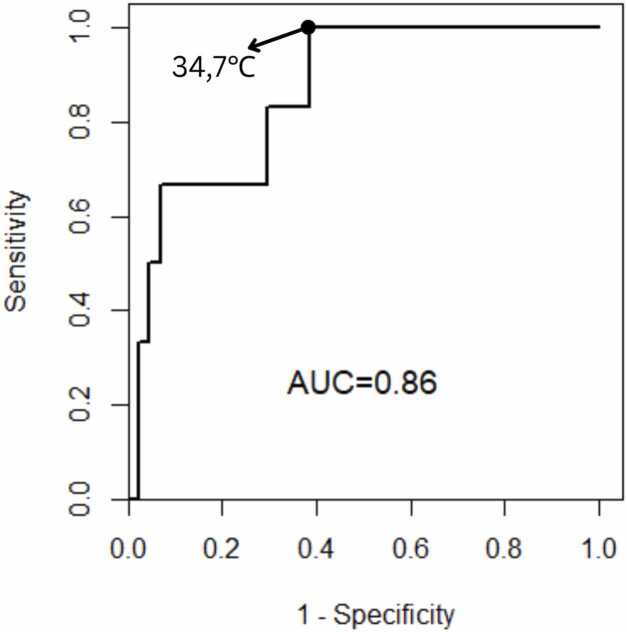
*Receiver operating characteristic (ROC) curve of peri-driveline temperature for detection of driveline infection.* The apparent AUC was 0.860 (95% CI 0.717-1.000). The marked point indicates the exploratory threshold of 34.7°C. At this threshold, no false negatives were observed, with sensitivity 100.0% and specificity 61.4%.

Peri-driveline temperature yielded an apparent AUC of 0.860 (95% CI 0.717-1.000; Figure 4) for DLI. The exploratory threshold was 34.7°C, at which no false negatives were observed. This yielded sensitivity 100.0% (95% CI 54.1-100.0), specificity 61.4% (95% CI 45.5-75.6), PPV 26.1% (95% CI 10.2-48.4), and NPV 100.0% (95% CI 87.2-100.0) (Table 3). At this threshold, there were 6 true positives, 0 false negatives, 27 true negatives, and 17 false positives. Among these false-positive patients, 8/17 were receiving antibiotics at imaging. Among the 13 false-positive patients with available hs-CRP, hs-CRP was generally low or mildly elevated, with a median of 3.0 mg/L [IQR 1.5-6.0] (Supplementary Table 7).

**Table 3:** ROC Analysis Data Summary Table

Cut-off (°C)	AUC (95% CI)	Sensitivity (95% CI)	Specificity (95% CI)	PPV (95% CI)	NPV (95% CI)
34.7	0.86 (0.86-1.00)	1.00 (0.54-1.00)	0.61 (0.45-0.76)	0.26 (0.10-0.48)	1.00 (0.87-1.00)

AUC, Area Under the Curve; CI, Confidence Interval; NPV, Negative Predictive Value; PPV, Positive Predictive Value; ROC, Receiver Operating Characteristic.

In stratified bootstrap internal stability assessment, the median AUC across 2000 resamples was 0.864 (95% percentile interval 0.712-0.977). The bootstrap-selected threshold varied from 34.668°C to 36.168°C, and sequential exclusion of each DLI episode yielded AUCs ranging from 0.836 to 0.909, supporting internal stability of the discriminatory signal while confirming that the exact threshold remains exploratory (Supplementary Table 4).

### Predictors of DLI

In univariable logistic regression, each 1.0°C increase was associated with higher odds of DLI (OR 4.55; *p* = 0.017). In univariable Firth regression, the association remained significant (OR 3.73, 95% CI 1.37-14.46; *p* = 0.006).

In the exploratory multivariable Firth regression including hs-CRP and peri-driveline-temperature (*n* = 46 with hs-CRP available), peri-driveline temperature remained associated with DLI after accounting for hs-CRP (Table 4). Temperature and hs-CRP were weakly correlated (ρ = 0.11; *p* = 0.45). However, given the small number of DLI events, these estimates should be interpreted as exploratory and event-limited rather than confirmatory.

**Table 4:** Logistic Regression Results

Method	**Predictor**	**OR (95% CI)**	***p*-value**
Univariable Logistic Regression	Peri-driveline temperature	4.55 (1.57-20.15)	0.017
Firth Logistic Regression	Peri-driveline temperature	3.73 (1.37-14.5)	0.006
Multivariable Firth Regression	Peri-driveline temperature	4.62 (1.49-25.9)	0.004
	hs-CRP	1.03 (1.00-1.06)	0.035

CI, Confidence Intervals; hs-CRP, high-sensitivity C-Reactive Protein; OR, Odds Ratio.

In exploratory analyses, peri-driveline temperature was not materially associated with age, sex, or BMI category (Supplementary Table 8).

### Sensitivity analysis

Including the second infection admission (*n* = 51; 7 DLI) produced a similar AUC (0.86; 95% CI 0.73-0.99) and the same threshold (34.7°C). Sensitivity, specificity, and NPV were unchanged, while PPV increased modestly (to 29%). These results are summarized in Supplementary Tables 1 and 2.

### Exploratory ΔT analysis

As an exploratory relative-temperature analysis, ΔT was calculated as peri-driveline ROI temperature minus the mean of 2 abdominal reference-skin ROIs. ΔT was higher in patients with DLI than in those without DLI: 0.459°C [IQR 0.418-0.512] versus 0.233°C [IQR 0.100-0.414] (exact Wilcoxon *p* = 0.016). The apparent AUC was 0.799 (95% CI 0.669-0.929). At the exploratory threshold of 0.321°C, no false negatives were observed, with moderate specificity; full diagnostic metrics and bootstrap results are shown in Supplementary Table 5. In the episode-level sensitivity analysis including 1 additional infected episode, the ΔT signal was attenuated, with an apparent AUC of 0.701 (95% CI 0.478-0.924) and 1 false-negative result at the primary ΔT threshold.

### ROI consistency assessment

Repeated blinded driveline ROI placement by the same reader showed low intra-reader variability, with a mean absolute difference of 0.059°C, median absolute difference of 0.044°C [IQR 0.016-0.074], maximum absolute difference of 0.342°C, and no differences >1.0°C (Supplementary Table 9).

## Discussion

In this prospective diagnostic-accuracy pilot study, handheld infrared thermography distinguished LVAD patients with and without DLI with good apparent discrimination. Peri-driveline temperature was higher in DLI compared with non-DLI, and at the exploratory threshold, no false negatives were observed in this small pilot cohort. However, the confidence interval around sensitivity was wide, specificity was moderate, and the threshold was derived and tested in the same dataset. The bootstrap and influence analyses should be interpreted as internal stability assessments only and not as validation, as validation would require testing a prespecified threshold in an independent cohort. These findings should therefore be interpreted as hypothesis-generating rather than clinically validated.

As a secondary exploratory analysis, ΔT was evaluated to assess relative peri-driveline warmth compared with abdominal reference skin. ΔT was higher in DLI-positive than DLI-negative patients and showed apparent discriminatory performance, but the signal was attenuated in the episode-level sensitivity analysis, including 1 additional infected episode. Because this analysis was non-prespecified, reference-skin ROI placement was exploratory, and the ΔT threshold was derived within the same small dataset, these findings should be interpreted as hypothesis-generating only.

### Clinical interpretation and potential role in care

With the presently available data, thermography is best viewed as a rapid, non-invasive adjunct for triage rather than as a stand-alone diagnostic test for LVAD DLI. In this pilot study, imaging was performed in a pragmatic clinical workflow, including routine outpatient assessments and hospital admissions for DLI. Its most plausible clinical role is in patients with suspected infection or equivocal local findings, where a low-threshold thermal signal may support earlier recognition and closer evaluation. However, the moderate specificity observed in this cohort means that elevated peri-driveline temperature should not be considered confirmatory. A positive thermographic result should instead prompt focused clinical review and, where indicated, additional DLI work-up, including exit-site assessment, wound or exit-site culture, hs-CRP and other laboratory testing, repeat assessment, and imaging when deeper driveline, pump, or systemic infection is suspected.^24^, ^25^ Thermography alone should not guide antibiotic initiation; treatment decisions should remain based on the overall clinical assessment and established LVAD infection care pathways. No clinical implementation threshold should be inferred from this study until multicenter validation using prespecified thresholds has been completed.

### Comparison with existing evidence

Our findings are consistent with the broader thermography literature in infectious and inflammatory conditions, where local skin temperature elevation provides a sensitive signal of inflammation but does not reliably discriminate infection from non-infectious inflammatory states. In studies of cellulitis, thermal imaging has consistently demonstrated high sensitivity but limited specificity when used in isolation, reflecting overlap between infectious and non-infectious inflammation.^14^, ^15^, ^16^ Similarly, wound-monitoring studies show that thermography can detect early inflammatory changes, but that temperature elevation alone is insufficient to confirm infection, supporting its role as a complementary rather than definitive diagnostic tool.^12^, ^17^, ^18^, ^19^

The present study extends these observations to the LVAD population by providing pilot quantitative, temperature-derived diagnostic accuracy estimates for DLI in a real-world clinical cohort.

### Why specificity may be limited

Several mechanisms may explain false-positive temperature elevations around the driveline. The exit site is prone to chronic mechanical stress, micro-trauma, and dressing-related irritation, including contact dermatitis, which can produce sterile inflammation with localized hyperemia and an increased thermal signal that may mimic infection.^17^, ^18^, ^25^, ^26^ Observational evidence also suggests that contact dermatitis is associated with a higher incidence of DLI, implying that inflammatory skin changes may both confound thermographic interpretation and reflect a clinical state with increased susceptibility to infection.^27^ In the present study, however, dermatitis, recent manipulation, dressing-related irritation, and other local non-DLI causes of warmth were not prospectively captured in a standardized manner, so these mechanisms should be interpreted as plausible explanations rather than confirmed causes of false-positive results. Technical factors may additionally contribute, including variation in camera distance/angle, incomplete exclusion of non-skin regions, and residual artifact signal within the ROI, which can bias temperature estimates upward. Collectively, these findings support using thermography as an adjunct interpreted alongside clinical assessment rather than a stand-alone diagnostic test.

### Limitations

This study has several limitations that affect generalizability. First, the diagnostic threshold was derived and evaluated within the same dataset, introducing optimism bias and inflating apparent performance. Although bootstrap and sequential exclusion of each DLI episode analysis supported internal stability of the discriminatory signal, they do not constitute external validation, and the exact threshold remains exploratory. Second, the number of DLI events was small, resulting in imprecise performance estimates and limiting stable multivariable modeling; although Firth’s penalized logistic regression was used to reduce small-sample bias, all effect estimates should be considered exploratory. Third, the primary reference diagnosis remained the treating team’s final clinical diagnosis, although DLI-positive episodes were retrospectively mapped to ISHLT-compatible categories. Fourth, although image analysis was performed on anonymized files, complete blinding cannot be guaranteed because contextual cues may occasionally suggest infection status. In addition, systematic body thermometer measurements were not obtained, so the association between peri-driveline temperature and measured body temperature could not be assessed. The ΔT analysis used abdominal reference-skin ROIs as relative skin-temperature comparators, not as a substitute for measured body temperature. Because ambient temperature, exact camera angle, exact camera-to-skin distance, and patient activity before imaging were not systematically recorded, both absolute temperature values and ΔT thresholds may be influenced by acquisition conditions. The ΔT threshold should therefore be considered exploratory and not validated. This pilot study did not evaluate downstream testing decisions, resource utilization, net clinical benefit, or patient outcomes; therefore, clinical utility remains unproven. Finally, temperature calibration was image-specific and based on displayed minimum and maximum values, enabling consistent within-image extraction but not external validation of absolute temperature accuracy.

Future studies should include external multicenter validation with a pre-specified temperature threshold and standardized acquisition conditions.

Larger prospective studies should evaluate whether thermography adds diagnostic value to a prespecified clinical-assessment pathway incorporating local signs, microbiology, hs-CRP, and imaging when indicated. Such studies should also assess downstream testing impact, resource utilization, net benefit, and patient outcomes.

## Conclusions

In this prospective pilot cohort, handheld infrared thermography showed promising apparent discrimination for LVAD DLI. At the exploratory threshold, no false negatives were observed, but specificity was moderate and performance estimates were imprecise because of the small number of DLI events. Our findings suggest that thermography may have value as an adjunctive triage marker to support timely clinical evaluation of suspected DLI, but it should not be used as a stand-alone diagnostic test or as a threshold for antibiotic initiation. Larger multicenter studies are required to support these findings and to enable validation using pre-specified thresholds, standardized definitions, and assessment of reproducibility prior to clinical implementation.

## Data Availability Statement

The data supporting the findings of this study are available from the corresponding author upon reasonable request.

## Disclosure statement

KD reports speaker and consultancy fees to his institution from Boehringer Ingelheim, AstraZeneca, Abbott, FIRE1, and Echosense. JMtM reports speaker and/or consultancy fees to her institution from Novartis, Bayer, Boehringer Ingelheim, Johnson & Johnson, Roche, and Novo Nordisk and receives grants from the Dutch Heart Foundation and Netherlands Organization for Scientific Research (NWO) outside the submitted work.

## Declaration of Generative AI and AI-Assisted Technologies in the Writing Process

During the preparation of this work, the author(s) used ChatGPT to screen part of the available bibliography. After using this tool/service, the author(s) reviewed and edited the content as needed and take(s) full responsibility for the content of the published article.

## Declaration of Competing Interest

The authors declare the following financial interests/relationships, which may be considered as potential competing interests: K. Damman reports consultancy and speaker fees to his institution by Boehringer Ingelheim Pharmaceuticals Inc, AstraZeneca, Abbott, FIRE1 and Echosense. JJ. ter Maaten reports consultancy and speaker fees to her institution by Novartis Pharmaceuticals Corporation, Bayer Corporation, Boehringer Ingelheim Ltd, Johnson & Johnson MedTech, Roche, and Novo Nordisk. JJ. ter Maaten is supported by a grant of the Dutch Heart Foundation and Dutch Research Council. All other authors, declare that they have no known competing financial interests or personal relationships that could have appeared to influence the work reported in this paper.

## Footnotes

Appendix A Supplementary data associated with this article can be found in the online version at doi:10.1016/j.jhlto.2026.100613.
