# Supplementary material for: Handheld infrared thermography for triage of LVAD driveline infection: A prospective pilot diagnostic accuracy study
Source: JHLT Open. 2026 Jun 15;13:100613. doi: 10.1016/j.jhlto.2026.100613 (PMC13401013; doi:10.1016/j.jhlto.2026.100613)
Supplement: Supplementary file 1 — Supplementary material [file mmc1.docx]

**Supplement**:

**Supplementary Table 1.** Sensitivity Analysis: ROC Curve, AUC, and Diagnostic Indices

|  | **Cut-off (°C)** | **AUC (95% CI)** | **Sensitivity (95% CI)** | **Specificity (95% CI)** | **PPV (95% CI)** | **NPV (95% CI)** |
| --- | --- | --- | --- | --- | --- | --- |
| **Sensitivity Analysis** | 34.7 | 0.86 (0.86-0.99) | 1.00 (0.59- 1.00) | 0.61 (0.45-0.76) | 0.29 (0.13-0.51) | 1.00 (0.87-1.00) |

AUC, Area Under the Curve; CI, Confidence Interval; NPV, Negative Predictive Value; PPV, Positive Predictive Value; ROC, Receiver Operating Characteristic.

**Supplementary Table 2.** Sensitivity Analysis Logistic Regressions results

|  | **Predictor** | **OR** | **95% CI** | **p-value** |
| --- | --- | --- | --- | --- |
| **Univariable Logistic Regression** | Peri-driveline temperature | 4.66 | 1.66 - 19.38 | 0.012 |
| **Firth Logistic Regression** | Peri-driveline temperature | 3.86 | 1.46 - 14.31 | 0.003 |
| **Multivariable Firth Regression** | Peri-driveline temperature | 4.63 | 1.57 - 23.12 | 0.002 |
|  | hs-CRP | 1.03 | 1.00 - 1.06 | 0.038 |

CI; Confidence Intervals, hs-CRP; high sensitivity C-Reactive Protein, OR; Odds Ratio

Supplementary Table 3. Retrospective ISHLT compatible adjudication of DLI-positive episodes

| **Episode / Patient Study ID** | **Evidence supporting percutaneous driveline/lead infection** | **Complicating ISHLT features assessed** | **Final ISHLT-compatible classification and rationale** |
| --- | --- | --- | --- |
| 01 / Patient ID 01 | **Local signs:** erythema, pain, tenderness, swelling, purulent exit-site discharge. **Exit-site/wound culture:** positive for S. aureus. | **Systemic signs:** none  **Blood** **cultures:**2x negative.  **hs-CRP at presentation:** 6 mg/L. **Imaging:** FDG-PET/CT: localized subcutaneous infection around driveline exit site; no driveline/LVAD extension; no metastatic infectious foci. **Missing data:** none | **Classification:** Uncomplicated percutaneous lead infection; localized driveline exit-site/subcutaneous involvement. **Rationale:** Local DLI with purulent discharge and positive S. aureus wound culture, without systemic signs, bacteremia, or PET/CT evidence of driveline/LVAD extension. |
| 02 / Patient ID 01 | **Local signs:** erythema, pain, tenderness, swelling, purulent exit-site discharge. **Exit-site/wound culture:** positive for S. aureus. | **Systemic signs:** none  **Blood** **cultures:**2x negative  **hs-CRP at presentation:** 56 mg/L.  **Imaging:** FDG-PET/CT: unchanged localized inflammatory changes at driveline exit site; no extension, LVAD involvement, or metastatic infectious foci. **Missing data:** none | **Classification:** Uncomplicated percutaneous lead infection; recurrent localized driveline exit-site infection treated with debridement/washout.  **Rationale:** Recurrent local DLI with positive S. aureus wound culture and no systemic or imaging evidence of deep/device involvement; wound improved after debridement/washout and IV flucloxacillin. |
| 03 / Patient ID 28 | **Local signs:** erythema. **Exit-site/wound culture:** not obtained. | **Systemic signs:** fever, sepsis, leukocytosis  **Blood** **cultures:** 2x negative  **hs-CRP at presentation:** 96 mg/L  **Imaging:** FDG-PET/CT: mild focal increased uptake in LVAD outflow graft/tract, suspicious for possible infection. **Missing data:** exit-site/wound culture not obtained. | **Classification:** Complicated percutaneous lead/MCS infection; possible LVAD outflow graft involvement. **Rationale:** Fever/sepsis, elevated hs-CRP, and PET/CT suspicion of outflow graft involvement support complicated infection rather than localized uncomplicated DLI. |
| 04 / Patient ID 39 | **Local signs:** erythema, pain, exit-site discharge. **Exit-site/wound culture:** staphylococcus aureus | **Systemic signs:** fever, leukocytosis  **Blood** **cultures:** 2x negative  **hs-CRP at presentation:** 3.8 mg/L  **Imaging:** FDG-PET/CT: Extensive driveline infection with intra-abdominal extension, bordering on intrathoracic involvement.  Surgical  **Missing data:** none | **Classification:** Complicated percutaneous lead/MCS infection; extensive intra-abdominal driveline tract infection approaching intrathoracic involvement.  **Rationale:** Positive S. aureus wound culture with fever/leukocytosis and PET/CT-confirmed extensive driveline extension supports complicated infection, despite negative blood cultures. |
| 05 / Patient ID 42 | **Local signs:** erythema, pain, swelling, purulent exit-site discharge  **Exit-site/wound culture:** pseudomonas aeruginosa | **Systemic signs:** leukocytosis  **Blood** **cultures:** 2x negative  **hs-CRP at presentation:** 34 mg/L  **Imaging:** FDG-PET/CT: active subcutaneous infection at the driveline exit site in the left hemiabdomen. Moderate FDG uptake along the intra-abdominal driveline segment, interpreted as probably reactive  **Missing data:** none | **Classification:** Complicated percutaneous lead/MCS infection; recurrent DLI with surgically treated exit-site involvement and possible intra-abdominal driveline involvement. **Rationale:** Recurrent DLI requiring debridement/washout and exit-site relocation, with PET/CT showing active subcutaneous exit-site infection and moderate intra-abdominal driveline uptake, supports complicated infection rather than localized uncomplicated DLI. |
| 06 / Patient ID 48 | **Local signs:** erythema, pain **Exit-site/wound culture:** negative | **Systemic signs:** none  **Blood** **cultures:** 2x negative  **hs-CRP at presentation:** 117 mg/L  **Imaging:** FDG-PET/CT: progression of the known driveline infection with intrathoracic extension toward the LVAD and, to a lesser extent, the proximal outflow graft/tract.  **Missing data:** none | **Classification:** Complicated percutaneous lead/MCS infection; intrathoracic driveline extension toward the LVAD and proximal outflow graft/tract. **Rationale:** PET/CT showed progression of known driveline infection with intrathoracic extension toward the LVAD and proximal outflow graft/tract, supporting complicated infection despite negative exit-site and blood cultures and absence of systemic signs. |
| 07 / Patient ID 19 | **Local signs:** pain **Exit-site/wound culture:** staphylococcus aureus | **Systemic signs:** none  **Blood** **cultures:** 2x negative  **hs-CRP at presentation:** 11 mg/L  **Imaging:** FDG-PET/CT: superficial driveline infection extending 8 cm subcutaneously; no LVAD involvement, no metastatic infectious foci, and no systemic inflammatory signs.  **Missing data:** none | **Classification:** Complicated percutaneous lead infection; superficial subcutaneous driveline extension without LVAD involvement. **Rationale:** Positive S. aureus exit-site culture and PET/CT-confirmed 8-cm subcutaneous driveline extension support complicated infection despite negative blood cultures and no systemic/device involvement. |

DLI, driveline infection; FDG-PET/CT, fluorodeoxyglucose positron emission tomography/computed tomography; hs-CRP, high-sensitivity C-reactive protein; ISHLT, International Society for Heart and Lung Transplantation; LVAD, left ventricular assist device; MCS, mechanical circulatory support.

**Supplementary Table 4.** Internal stability assessment of ROC-derived AUC and Youden threshold for mean peri-driveline temperature

| **Analysis / metric** | **Estimate** | **95% CI / percentile interval / range** |
| --- | --- | --- |
| **Primary apparent ROC analysis** | | |
| AUC | 0.860 | 0.717-1.000 |
| Youden-derived threshold | 34.682 °C | - |
| Sensitivity at apparent threshold | 100.0% | 54.1-100.0% |
| Specificity at apparent threshold | 61.4% | 45.5-75.6% |
| PPV at apparent threshold | 26.1% | 10.2-48.4% |
| NPV at apparent threshold | 100.0% | 87.2-100.0% |
| Confusion matrix at apparent threshold | TP 6, FN 0, TN 27, FP 17 | - |
| **Stratified bootstrap stability assessment, 2000 resamples** | | |
| Bootstrap AUC | 0.864 | 0.712-0.977 |
| Bootstrap-selected Youden threshold | 34.866 °C | 34.668-36.168 °C |
| Sensitivity after applying bootstrap-selected thresholds to original data | 83.3% | 50.0-100.0% |
| Specificity after applying bootstrap-selected thresholds to original data | 70.5% | 59.1-95.5% |
| PPV after applying bootstrap-selected thresholds to original data | 27.8% | 25.0-60.0% |
| NPV after applying bootstrap-selected thresholds to original data | 96.8% | 93.3-100.0% |
| **Sequential exclusion of each DLI episode** | | |
| AUC range | - | 0.836-0.909 |
| Youden threshold range | - | 34.682-35.859 °C |

AUC, area under the curve; CI, confidence interval; DLI, driveline infection; FN, false negative; FP, false positive; NPV, negative predictive value; PPV, positive predictive value; ROC, receiver operating characteristic; TN, true negative; TP, true positive; °C, degrees Celsius.

**Supplementary Table 5.** Exploratory ΔT analysis using abdominal reference-skin ROIs.

| **Measure** | **Primary patient-level ΔT analysis** | **Stratified bootstrap internal stability** | **Episode-level sensitivity analysis** |
| --- | --- | --- | --- |
| Cohort | N = 50 | N = 50 | N = 51 |
| DLI-positive | 6 patients | 6 patients | 7 episodes |
| DLI-negative | 44 patients | 44 patients | 44 episodes |
| ΔT in DLI-positive | 0.459°C [0.418-0.512] | - | 0.441°C [0.367-0.501] |
| ΔT in DLI-negative | 0.233°C [0.100-0.414] | - | 0.233°C [0.100-0.414] |
| Exact Wilcoxon p-value | 0.016 | - | 0.093 |
| AUC (95% CI / percentile interval) | 0.799 (0.669-0.929) | 0.803 median (0.663-0.917) | 0.701 (0.478-0.924) |
| ΔT threshold | 0.321°C | 0.321°C median (0.314-0.451) | 0.321°C |
| Sensitivity (95% CI) | 100.0% (54.1-100.0) | - | 85.7% (42.1-99.6) |
| Specificity (95% CI) | 65.9% (50.1-79.5) | - | 65.9% (50.1-79.5) |
| PPV (95% CI) | 28.6% (11.3-52.2) | - | 28.6% (11.3-52.2) |
| NPV (95% CI) | 100.0% (88.1-100.0) | - | 96.7% (82.8-99.9) |
| Confusion matrix | TP 6, FP 15, FN 0, TN 29 | - | TP 6, FP 15, FN 1, TN 29 |

AUC, area under the receiver operating characteristic curve; CI, confidence interval; DLI, driveline infection; FN, false negative; FP, false positive; IQR, interquartile range; NPV, negative predictive value; PPV, positive predictive value; ROI, region of interest; TN, true negative; TP, true positive.

**Supplementary Table 6.** Antibiotic exposure, indication, and type by DLI status

| **Variable** | **DLI-positive, n=6** | **DLI-negative, n=44** |
| --- | --- | --- |
| **Antibiotic exposure at imaging** | | |
| Antibiotics at imaging | 6/6 (100.0%) | 11/44 (25.0%) |
| No antibiotics at imaging | 0/6 (0.0%) | 33/44 (75.0%) |
| **Antibiotic indication** | | |
| No antibiotics | 0 | 33 |
| DLI | 6 | 0 |
| Post-infection treatment | 0 | 1 |
| Chronic suppressive therapy | 0 | 8 |
| Prophylaxis until HTx | 0 | 2 |
| **Antibiotic type** | | |
| Flucloxacillin IV | 4 | 0 |
| Flucloxacillin oral | 2 | 0 |
| Ceftazidime | 0 | 0 |
| Clindamycin | 0 | 3 |
| Augmentin | 0 | 1 |
| Cefalexin / cefalexine | 0 | 2 |
| Ciprofloxacin | 0 | 1 |
| Doxycycline | 0 | 2 |
| Minocycline | 0 | 2 |

DLI, driveline infection; HTx, heart transplantation; IV, intravenous; n/N, number/total number.

**Supplementary Table 7.** Exploratory DLI-negative subgroup characteristics by antibiotic exposure at imaging

| **Variable** | **DLI-negative without antibiotics, n=33** | **DLI-negative with antibiotics, n=11** | **p-value** |
| --- | --- | --- | --- |
| Peri-driveline temperature, °C | 34.27 [33.77-34.84] | 34.86 [34.39-35.34] | 0.074 |
| hs-CRP, mg/L | 3.0 [1.35-7.5] (n=31) | 4.8 [2.2-9.0] (n=9) | 0.496 |
| ΔT, °C | 0.190 [0.074-0.394] | 0.399 [0.212-0.477] | 0.122 |
| Test-positive at absolute-temperature threshold | 9/33 (27.3%) | 8/11 (72.7%) | 0.012 |

DLI, driveline infection; hs-CRP, high-sensitivity C-reactive protein; ΔT, peri-driveline temperature minus abdominal reference-skin temperature; °C, degrees Celsius; mg/L, milligrams per liter; n, number

**Supplementary Table 8.** Assessment of peri-driveline temperature according to age, sex, and BMI

| **Cohort** | **Variable** | **Comparison / analysis** | **n** | **Result** | **p-value** |
| --- | --- | --- | --- | --- | --- |
| **Overall cohort** | Age | Continuous | 50 | Spearman ρ = 0.147 | 0.309 |
|  | Age | <50 years | 12 | 34.29 [33.98-35.14] | 0.609 |
|  | Age | ≥50 years | 38 | 34.68 [33.94-35.26] |  |
|  | Sex | Female | 10 | 34.55 [33.90-35.23] | 0.636 |
|  | Sex | Male | 40 | 34.66 [33.95-35.28] |  |
|  | BMI | Continuous | 50 | Spearman ρ = 0.084 | 0.562 |
|  | BMI | <30 kg/m2 | 35 | 34.67 [33.92-35.15] | 0.882 |
|  | BMI | ≥30 kg/m2 | 15 | 34.34 [33.99-35.40] |  |
| **DLI-negative patients** | Age | Continuous | 44 | Spearman ρ = 0.164 | 0.289 |
|  | Age | <50 years | 11 | 34.17 [33.95-34.83] | 0.665 |
|  | Age | ≥50 years | 33 | 34.48 [33.88-35.12] |  |
|  | Sex | Female | 10 | 34.55 [33.90-35.23] | 0.966 |
|  | Sex | Male | 34 | 34.38 [33.91-34.97] |  |
|  | BMI | Continuous | 44 | Spearman ρ = -0.004 | 0.982 |
|  | BMI | <30 kg/m2 | 31 | 34.64 [33.89-35.07] | 0.571 |
|  | BMI | ≥30 kg/m2 | 13 | 34.17 [33.96-35.00] |  |

BMI, body mass index; DLI, driveline infection; IQR, interquartile range; kg/m², kilograms per square meter; n, number; ρ, Spearman’s rank correlation coefficient; °C, degrees Celsius.

**Supplementary Table 9.** Intra-reader consistency of repeated peri-driveline ROI placement

| **Metric** | **Result** |
| --- | --- |
| Assessment | Repeated blinded peri-driveline ROI placement by the same reader |
| ROI assessed | Peri-driveline skin ROI |
| Images/measurements assessed | n = 52 |
| Mean absolute difference | 0.059 °C |
| Median absolute difference | 0.044 °C |
| IQR absolute difference | 0.016-0.074 °C |
| Maximum absolute difference | 0.342 °C |
| Repeated measurements differing by >1.0 °C | 0/52 (0.0%) |

IQR, interquartile range; n, number; ROI, region of interest; °C, degrees Celsi

**Thermal imager details:**

| **Variable** | **Specifications** |
| --- | --- |
| Device model | Lodestar LTi120S Infrared Thermal Imager |
| Intended use | Surface temperature measurement |
| Operational temperature range | -20 to 400 °C |
| Measurement accuracy | ± 2% of the measured value |
| Thermal detector resolution | 120 × 90 pixels |
| Visual (RGB) image resolution | 320 × 240 pixels |
| Spectral range | 8-14 µm |
| Thermal sensitivity (NETD) | 60 mK |

°C, degrees Celsius; mK, millikelvin; NETD, noise-equivalent temperature difference; RGB, red-green-blue; µm, micrometre.
